# Checklist of the flower flies of Ecuador (Diptera, Syrphidae)

**DOI:** 10.3897/zookeys.691.13328

**Published:** 2017-08-17

**Authors:** Diego Marín-Armijos, Noelia Quezada-Ríos, Carolina Soto-Armijos, Ximo Mengual

**Affiliations:** 1 Museo de Colecciones Biológicas, Departamento de Ciencias Biológicas, Universidad Técnica Particular de Loja, San Cayetano Alto s/n, C.P. 11 01 608, Loja, Ecuador; 2 Zoologisches Forschungsmuseum Alexander Koenig, Leibniz-Institut für Biodiversität der Tiere, Adenauerallee 160, D-53113 Bonn, Germany

**Keywords:** faunistics, hoverflies, Neotropical Region, species list, Syrphid fauna

## Abstract

Syrphidae is one of the most speciose families of true flies, with more than 6,100 described species and worldwide distribution. They are important for humans acting as crucial pollinators, biological control agents, decomposers, and bioindicators. One third of its diversity is found in the Neotropical Region, but the taxonomic knowledge for this region is incomplete. Thus, taxonomic revisions and species checklists of Syrphidae in the Neotropics are the highest priority for biodiversity studies. Therefore, we present the first checklist of Syrphidae for Ecuador based on literature records, and provide as well the original reference for the first time species citations for the country. A total of 201 species were recorded for Ecuador, with more than 600 records from 24 provinces and 237 localities. Tungurahua, Pastaza, and Galápagos were the best sampled provinces. Although the reported Ecuadorian syrphid fauna only comprises 11.2 % of the described Neotropical species, Ecuador has the third highest flower fly diversity density after Costa Rica and Suriname. These data indicate the high species diversity for this country in such small geographic area.

## Introduction

Seventeen countries in the world are considered megadiverse, occupying less than 10% of the Earth’s surface and comprising nearly 70% of the global biodiversity (Mittermeier et al. 2005). In this group, Ecuador is listed among the first places in the world ranking based on number of species per area unit for vascular plants, mammals, birds, reptiles and amphibians (Mittermeier and Mittermeier 1997, Brehm et al. 2008), but it is the megadiverse country with the smallest land area (Mittermeier et al. 2005). This makes Ecuador rank at the top, or near so, of the megadiversity list if diversity per unit area is taken into consideration. The geographic position of Ecuador and a series of biotic and abiotic elements have resulted in an extraordinary biological diversity in this nation. For instance, there are ca. 20,000 estimated plants in Ecuador, of which up to 5,000 are most likely endemics. In terms of vertebrate diversity, 1.3% of the global diversity of non-fish vertebrates is endemic to Ecuador (Mittermeier et al. 2005).

Insects are the most successful group of living organisms in our planet in number of species and different natural histories. From all known and described species on Earth, *ca.* 1.5–1.7 millions, between 65 and 75% are insects, and among the insect orders only four orders represent more than 80% of the species: Coleoptera, Lepidoptera, Diptera, and Hymenoptera (Adler and Foottit 2009). Despite being abundant and ubiquitous, insects are understudied in Ecuador and many new species and genera are waiting to be formally described (Dangles et al. 2009, Barragán et al. 2009). Furthermore, there is no estimate on the number of invertebrates for Ecuador, neither a checklist for most of the invertebrate groups present in this country (Salazar and Donoso 2014).

Diptera, which includes mosquitoes and true flies, comprises more than 153,000 described species (about 10–12% of the planet’s biodiversity) and has a rate of near 1,000 new species described per year (Pape and Thompson 2013). Diptera is not only rich in number of species, but also in morphology and structure, habitats use and human interactions (Courtney et al. 2009). Most recent studies in this country have focused mostly on Lepidoptera (Piñas and Manzano 1997, Bollino and Onore 2001, Piñas and Manzano 2003a, 2003b, Hilt and Fiedler 2005, Brehm 2005, Fiedler et al. 2007, Bodner et al. 2010), and Coleoptera (Celi et al. 2004, Moret 2005, Carpio et al. 2009, Carvajal et al. 2011, Domínguez et al. 2015, Thormann et al. 2016). Salazar and Donoso (2014) present some numbers for the invertebrate fauna of Ecuador and report 722 dipteran species from the literature, but this number is probably an underestimation, which was biased by the research focus of the previous works in Ecuador. Thus, the actual species number of species of Diptera for the country is far from being known.

Commonly called flower or hoverflies, the family Syrphidae is one of the most diverse in Diptera with more than 6,000 described species (Brown 2009), and the third taxon with most species in the Neotropical Region (Amorim 2009). Their coloration, from orange-brown in a few species to striking yellow and black patterns, causes them to be confused with bees or wasps (Hymenoptera). Adults of the family Syrphidae have the ability to hover and are associated with flowers, which are used as mating sites and food sources (pollen and nectar). Therefore, the imagoes are considered important pollinators of herbs, shrubs, and arboreal plants in natural ecosystems as well as in agricultural areas (Speight and Lucas 1992, Marinoni and Thompson 2003, Pérez-Bañón et al. 2003, Ssymank and Kearns 2009, Inouye et al. 2015). Syrphid species have been used as bioindicators as well to assess biodiversity loss and the efficiency of restoration and conservation policies (Sommaggio 1999, Tscharntke et al. 2005, Ricarte et al. 2011, Sommaggio and Burgio 2014).

Larvae are very variable in structure, habits, and feeding modes, including fungal fruiting bodies, brood in nests of social Hymenoptera, dung, decaying wood and water bodies of several types (Rotheray 1993, Rotheray and Gilbert 1999, 2011). Larvae of some species can mine leaves and stems of numerous plant families, or even feed on pollen, and others are predators of arthropods (aphids, caterpillars, larvae of flies or beetles, adult flies, etc.) or are kleptoparasitic or parasitoids (Rojo et al. 2003, Weng and Rotheray 2008, Reemer and Rotheray 2009, Rotheray et al. 2000, Ureña and Hanson 2010, Zuijen and Nishida 2011, Pérez-Lachaud et al 2014, Jordaens et al. 2015, Fleischmann et al. 2016, Dumbardon-Martial 2016). Due to their feeding mode, some syrphid species play an important role as biological control agents of pests (Greco 1998, Schmidt et al. 2004, Bergh and Short 2008, Bugg et al. 2008, Pineda and Marcos-García 2008, Nelson et al. 2012, Amorós-Jiménez et al. 2014, Eckberg et al. 2015) and as decomposers of organic matter (Lardé 1989, Rotheray et al. 2009, Martínez-Falcón et al. 2012).

Flower flies are distributed worldwide, with the exception of Antarctica and a few remote oceanic islands, and their greatest species diversity is found in the tropics (Vockeroth 1992, Reemer 2013, Reemer and Ståhls 2013b). The classification of Neotropical Syrphidae has been largely reviewed by Vockeroth (1969), Thompson (1972, 1999) and Reemer (2014), but the taxonomy of Syrphidae is far from being complete in the Neotropical Region yet, and many new species remain to be described (Mengual and Thompson 2008, Mengual et al. 2009, Thompson et al. 2010, Mengual 2011, Mengual and Thompson 2011, Reemer 2010, 2014, 2016). Moreover, the almost absence of identification keys for Neotropical species makes difficult the elaboration of regional checklists or the discovery of new species to science (Thompson et al. 2010, Montoya et al. 2012). Previously, species lists based on single surveys (Campos 1960, Linsley and Usinger 1966, Linsey 1977), for specific taxa (genus *Toxomerus* Macquart, 1855 by Gerdes, 1974a), or for limited areas, such as Galapagos Islands (Sinclair 2015, Sinclair et al. 2016), have been published for Ecuador.

Amorim (2009) considers Syrphidae among the still underexplored dipteran families in the Neotropical Region, and Ecuador among the most poorly collected areas in South America. Nevertheless, there have been some efforts during the last years to teach Syrphidae taxonomy to young students via workshops and courses with the great help of F.C. Thompson (USNM, National Museum of Natural History, Smithsonian Institution) as a coordinator (Colombia 2006, Peru 2008, Ecuador 2012), with the purpose to educate new taxonomists that may help to elucidate the thrilling evolutionary history of this group. As a fruit from these workshops, a strong collaboration among the authors was established years ago to study the flower flies of Ecuador. Currently, there is no species list for Ecuadorian flower flies that can help as a starting point, and the existing records are few and scattered thorough the literature. Consequently, a species checklist of the family Syrphidae in Ecuador for further biodiversity studies was the highest priority. In this survey, we present the first species checklist of Syrphidae for Ecuador based on literature records and provide as well the original reference for the first time species citations for the country of Ecuador.

## Materials and methods

Thompson et al. (1976) was used as the primary source to check for species cited previously for Ecuador. Based on that keystone publication and Thompson et al. (2010), we reviewed all the published literature up to date in order to find references to Ecuadorian syrphids. Moreover, bibliographic searches were performed in public and scientific journal databases such as Google Scholar, Scopus, ISI Web of Knowledge, BioOne, Redalyc, Scielo, BioOne, ScienceDirect, and ResearchGate. Our keywords in English and Spanish for the searches were invertebrates, Ecuador, Diptera, Syrphidae, Neotropics, distribution, flower flies and hoverflies. In addition, we studied representative collections of Ecuador, i.e. Museo de Zoología de la Pontificia Universidad Católica del Ecuador (QCAZ) and Museo de la Escuela Politécnica Nacional.

**Figure 1.**
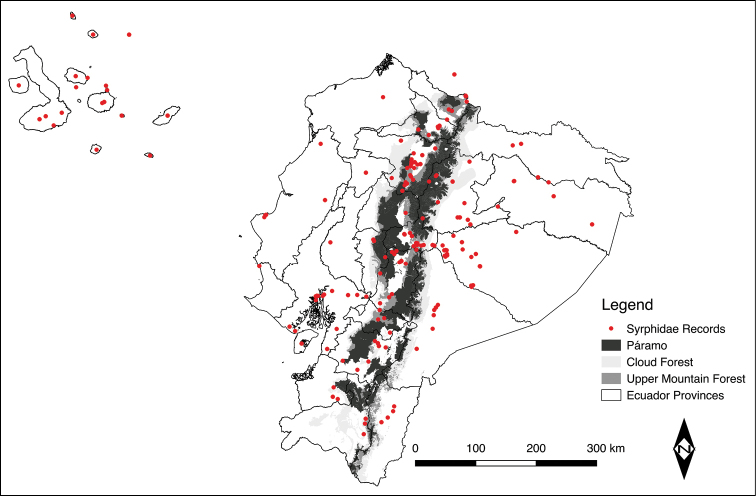
Distribution map of flower fly records in Ecuador.

To illustrate the flower fly records in a geographic map we used the coordinates available in the literature. For the localities without geographic coordinates we used Google Earth ® to obtain them. Figure 1 was created using QGIS software (QGIS Development Team, 2009).

## Results

A total of 201 species plus four unidentified species and two misidentified taxa, belonging to 51 syrphid genera and subgenera, have been recorded up to date for Ecuador. More than 600 records from 24 provinces and 237 different localities of Ecuador are given in Table 1. Although there are records from all the Ecuadorian provinces, they do not show an even collecting effort for the whole country. Tungurahua (with 80 collecting events), Pastaza (72), and Galápagos (60) are the best sampled provinces, while the flower fly records for Orellana (3), Los Ríos (2), Santa Elena (2), Santo Domingo de Los Tsáchilas (2), and Esmeraldas (1) provinces are almost anecdotal. In terms of geographic Ecuadorian regions, the Sierra of Ecuador and the Galapagos Islands have been more extensively sampled and studied (Table 1 and Figure 1). On the other hand, the Costa Region, North and South Amazonia, and Austral Region of Ecuador have been little explored (Figure 1).

**Table 1.** Checklist of Syrphidae species recorded from Ecuador, with the Ecuadorian province, locality, altitude (when cited in the original reference), and the original reference for Ecuador.

Species	Province	Locality (Altitude masl)	References for Ecuador
*Alipumilio femoratus* Shannon, 1927	Pastaza	Puyo	Rotheray et al. 2000: 137
*Allograpta annulipes* (Macquart, 1850)	Pastaza	Santa Inés, Río Pastaza (1200)	Fluke 1942: 16 (as *A. geminata*)
*Allograpta browni* Fluke, 1942	Imbabura	Cuicocha (3200)	Fluke 1942: 18
*Allograpta exotica* (Wiedemann, 1830)	Tungurahua	Baños	Fluke 1942: 19
	Tungurahua	Juive	Fluke 1942: 19
	Ecuador		Fluke 1950a: 146 (as *Syrphus exoticus*)
*Allograpta falcata* Fluke, 1942	Tungurahua	Baños (1800)	Fluke 1942: 16
	Tungurahua	Baños (2200)	Fluke 1942: 16
*Allograpta neosplendens* Sinclair & Thompson, 2016	Galápagos	Española	Sinclair and Peck 2002; Sinclair et al. 2016: 87
	Galápagos	Fernandina	Sinclair et al. 2016: 87
	Galápagos	Floreana	Coquillett 1901: 374; Linsley and Usinger 1966: 168; Linsley 1977: 39; Sinclair and Peck 2002; Sinclair 2015; Sinclair et al. 2016: 87
	Galápagos	Genovesa	Sinclair et al. 2016: 87
	Galápagos	Isabela	Sinclair and Peck 2002; Sinclair 2015; Sinclair et al. 2016: 87
	Galápagos	Marchena	Sinclair 2015
	Galápagos	Pinta	Sinclair and Peck 2002; Sinclair et al. 2016: 87
	Galápagos	San Cristóbal	Curran 1934: 153; Linsley and Usinger 1966: 168; Linsley 1977: 39; Sinclair and Peck 2002; Sinclair et al. 2016: 87
	Galápagos	Santa Cruz	Boada 2005: 84; Sinclair 2015; Sinclair et al. 2016: 87
	Galápagos	Santa Fé	Sinclair 2015
	Galápagos	Santiago	Coquillett 1901: 374; Curran 1934: 153; Linsley and Usinger 1966: 168; Linsley 1977: 39; Sinclair and Peck 2002; Sinclair et al. 2016: 87
	Galápagos		Thomson 1869: 501 (as *Syrphus splendens*)
*Allograpta neotropica* Curran, 1936	Tungurahua	Baños	Fluke 1942: 20
	Pastaza	Santa Inés	Fluke 1942: 20
*Allograpta obliqua* (Say, 1823)	Tungurahua	Baños	Fluke 1942: 19
	Carchi	Tulcán	Campos 1960: 25
	Chimborazo	Riobamba	Campos 1960: 25
*Allograpta tectiforma* Fluke, 1942	Imbabura	Cuicocha (3200)	Fluke 1942: 19
	Imbabura	Cuicocha (3500)	Fluke 1942: 19
	Ecuador		Fluke 1950a: 146
*Allograpta teligera* Fluke, 1942	Tungurahua	Baños (1800)	Fluke 1942: 18
*Argentinomyia agonis* (Walker, 1849)	Galápagos		Walker 1849: 588; Linsley 1977: 39
	Galápagos	Floreana	Sinclair and Peck 2002; Sinclair 2015
	Galápagos	Isabela	Sinclair and Peck 2002; Sinclair et al. 2016: 85
	Galápagos	Pinta	Sinclair and Peck 2002; Sinclair et al. 2016: 85
	Galápagos	San Cristóbal	Sinclair 2015
	Galápagos	Santa Cruz	Boada 2005: 86; Sinclair 2015; Sinclair et al. 2016: 85
*Argentinomyia altissima* (Fluke, 1945)	Imbabura	Cuicocha (3200)	Fluke 1945: 20
	Ecuador		Fluke 1958: 266
*Argentinomyia bolivariensis* (Fluke, 1945)	Bolívar	Hda. Talahua (3100)	Fluke 1945: 19
	Ecuador		Fluke 1958: 266
*Argentinomyia browni* (Fluke, 1945)	Bolívar	Hda. Talahua (3100)	Fluke 1945: 19
	Ecuador		Fluke 1958: 266
*Argentinomyia festiva* (Fluke, 1945)	Tungurahua	Baños (1800)	Fluke 1945: 10
*Argentinomyia longicornis* (Walker, 1836)	Pastaza	Puyo (3000)	Fluke 1945: 4
*Argentinomyia luculenta* (Fluke, 1945)	Tungurahua	Baños (2300)	Fluke 1945: 18
	Tungurahua	Pondoa (2800)	Fluke 1945: 18
*Argentinomyia opaca* (Fluke, 1945)	Chimborazo	Urbina (3650)	Fluke 1945: 11
	Ecuador		Fluke 1958: 266
*Argentinomyia rex* (Fluke, 1945)	Bolívar	Hda. Talahua (3100)	Fluke 1945: 22
	Ecuador		Fluke 1958: 266
*Argentinomyia tropica* (Curran, 1937)	Tungurahua	Baños (2300)	Fluke 1945: 17
*Claraplumula latifacies* Shannon, 1927	Bolívar	Hda. Talahua (3100)	Fluke 1942: 4
	Ecuador		Fluke 1950a: 146
Copestylum (Copestylum) otongaensis Rotheray & Hancock, 2007	Cotopaxi	Otonga	Rotheray et al. 2007: 290
Copestylum (Copestylum) tapia Rotheray & Hancock, 2007	Cotopaxi	Otonga	Rotheray et al. 2007: 307
Copestylum (Phalacromya) araceorum Ricarte & Rotheray, 2015	Cotopaxi	Otonga	Ricarte et al. 2015: 13
Copestylum (Phalacromya) beatricea (Hull, 1950)	Azuay	Cuenca (2650)	Fluke 1951b: 15 (as *Volucella ecuadorea*)
	Tungurahua	Baños	Fluke 1951b: 15 (as *Volucella ecuadorea*)
	Imbabura	Cuicocha (3300)	Fluke 1951b: 15 (as *Volucella ecuadorea*)
	Ecuador		Hull 1950: 236
Copestylum (Phalacromya) brunneum (Thunberg, 1789)	Chimborazo	Huigra	Campos 1960: 27 (as *Volucella esuriens*)
	Cañar	Azogues	Campos 1960: 27 (as *Volucella esuriens*)
Copestylum (Phalacromya) bulbosum (Fluke, 1951)	Tungurahua	Minza Chica (3750)	Fluke 1951b: 25
Copestylum (Phalacromya) camposi (Curran, 1939)	Guayas	Isla Puná, Puerto Grande	Curran 1939: 8
Copestylum (Phalacromya) chaetophorum (Williston, 1887)	Guayas	San Rafael	Campos 1960: 27
	Guayas	Bucay	Campos 1960: 27
Copestylum (Phalacromya) currani (Fluke, 1951)	Pichincha	Guayllabamba	Rotheray et al. 2009: 714
	Tungurahua	Baños	Fluke 1951b: 13
	Imbabura	Cuicocha (3300)	Fluke 1951b: 13
	Azuay	Cuenca (2650)	Fluke 1951b: 13
Copestylum (Phalacromya) fulvicorne (Bigot, 1883)	Guayas	San Eduardo	Campos 1960: 27
	Guayas	Durán	Campos 1960: 27
	Guayas	San Rafael	Campos 1960: 27
Copestylum (Phalacromya) hambletoni (Fluke, 1951)	Ecuador		Thompson et al. 1976: 77
Copestylum (Phalacromya) multipunctatum Rotheray & Hancock, 2009	Pichincha	Guayllabamba	Rotheray et al. 2009: 704
Copestylum (Phalacromya) nigripes (Bigot, 1857)	El Oro	Chillacocha	Campos 1960: 29 (as *Phalacromyia concolor*)
Copestylum (Phalacromya) placivum (Hull, 1943)	Pastaza	Santa Inés	Hull 1943b: 31
Copestylum (Phalacromya) rufoscutellare (Philippi, 1865)	Chimborazo	Mirador	Campos 1960: 29
Copestylum (Phalacromya) scintillans (Hull, 1949)	Galápagos	San Cristóbal (730)	Sinclair et al. 2016: 83
	Galápagos	Santa Cruz	Sinclair 2015 (as C. cf. viridana)
Copestylum (Phalacromya) sica (Curran, 1953)	Pichincha	Guayllabamba	Rotheray et al. 2009: 720
	Tungurahua	Baños	Curran 1953: 9
	Azuay	Tarqui	Curran 1953: 9
Copestylum (Phalacromya) splendens (Townsend, 1897)	Pichincha	Cotocollao	Campos 1960: 27 (as *Volucella opalina*)
	Tungurahua	Ambato	Campos 1960: 27 (as *Volucella opalina*)
	Loja	Loja	Campos 1960: 27 (as *Volucella opalina*)
	Chimborazo	Riobamba	Campos 1960: 27 (as *Volucella opalina*)
Copestylum (Phalacromya) viridigaster (Hull, 1943)	Ecuador		Hull 1943h: 41
Dasysyrphus aff. lotus (Williston, 1887)	Pichincha	Pichincha (3300)	Fluke 1942: 3
*Dolichogyna chilensis* (Walker, 1836)	Azuay	Narihuiña	Campos 1960: 29
*Dolichogyna mulleri* Fluke, 1951	Azuay	Girón	Fluke 1951a: 472
	Imbabura	Cuicocha (3200)	Fluke 1951a: 472
*Eosalpingogaster nigriventris* (Bigot, 1883)	Guayas	Isla Puná, Puerto Grande (253)	Fluke 1937: 11 (as *Salpingogaster liposeta*)
Eristalis (Eoseristalis) bogotensis Macquart, 1842	Ecuador		Thompson et al. 1976: 101
	Napo-Pichincha	Antisamilla to Pinatura (3350)	Thompson 1997: 223
	Pichincha	Santa Catalina Expt. Station (2780)	Thompson 1997: 223
	Pichincha	Quito (2850)	Thompson 1997: 223
Eristalis (Eoseristalis) bogotensis Macquart, 1842	Chimborazo	8 mi NE of Tixan	Thompson 1997: 223
	Chimborazo	Lago Zurucuchu	Thompson 1997: 223
	Tungurahua	Ambato (2700)	Thompson 1997: 223
	Carchi	Troya	Thompson 1997: 223
	Carchi	Tulcan (2800)	Thompson 1997: 223
	Carchi	El Ángel (2700)	Thompson 1997: 223
	Cañar	El Tambo (2800)	Thompson 1997: 223
	Pichincha	Pomasqui (2200)	Thompson 1997: 223
	Pichincha	Valle de Machachi (2900)	Thompson 1997: 223
	Loja	Loja (2500)	Thompson 1997: 223
	Azuay	Tarqui (2800)	Thompson 1997: 223
	Azuay	28 km S of Cuenca (2500–2800)	Thompson 1997: 223
	Azuay	Cuenca (2200)	Thompson 1997: 223
Eupeodes (Metasyrphus) rojasi Marneff, 1999	Azuay	Gualaduisa Road (2150)	Thompson 1999: 339
	Tungurahua	Baños	Thompson 1999: 339
	Bolívar	Chota River, Carchi (2000)	Thompson 1999: 339
	Imbabura	NW Ibarra, Taguando River (1650–1900)	Thompson 1999: 339
	Carchi	El Ángel (2700)	Thompson 1999: 339
	Carchi	10 km SW Tulcán (2900)	Thompson 1999: 339
	Imbabura	3km N Ibarra, Yaguarcocha (1950)	Thompson 1999: 339
	Pichincha	Pichincha, 2km W Cayambe (2300)	Thompson 1999: 339
*Fazia alta* (Curran, 1936)	Tungurahua	Baños	Fluke 1942: 14
	Tungurahua	Juive	Fluke 1942: 14
	Ecuador		Fluke 1950a: 146
*Fazia altissima* (Fluke, 1942)	Tungurahua	Volcán Tungurahua, Minza Chica (3200)	Fluke 1942: 10
	Tungurahua	Pondoa (2800)	Fluke 1942: 10
	Pichincha	Páramo del Cerro, Pasochoa (3300)	Fluke 1942: 10
	Pichincha	Hda. San Rafael, Río San Pedro (2700)	Fluke 1942: 10
	Pichincha	Uyumbicho (2650)	Fluke 1942: 10
	Pichincha	Hda. San Rafael (3000)	Fluke 1942: 10
	Imbabura	Cuicocha (3200)	Fluke 1942: 10
	Ecuador		Fluke 1950a: 145
	Pastaza	Puyo (1000)	Fluke 1942: 14
	Pichincha	Uyumbicho (2700)	Fluke 1942: 14
	Imbabura	Cuicocha (3200)	Fluke 1942: 14
*Fazia argentipila* (Fluke, 1942)	Tungurahua	Baños, Runtun trail (2100)	Fluke 1942: 14
	Pichincha	Hda. San Rafael (3000)	Fluke 1942: 14
	Azuay	Cuenca (2500)	Fluke 1942: 14
	Tungurahua	Baños, San Pablo (2200)	Fluke 1942: 14
	Bolívar	Hda. Talahua (3100)	Fluke 1942: 14
	Ecuador		Fluke 1950a: 145
*Fazia colombia* (Curran, 1925)	Tungurahua	Baños (2100)	Fluke 1942: 13
	Azuay	Cuenca (2500)	Fluke 1942: 13
	Pichincha	Pichincha (2700)	Fluke 1942: 13
	Morona Santiago	Sucúa (900)	Fluke 1942: 13
	Ecuador		Fluke 1950a: 146
*Fazia decemmaculata* (Shannon, 1927)	Ecuador		Thompson et al. 1976: 34 (as *F. bullaephora*); Mengual et al. 2009: 17 (as *F. bullaephora*)
*Fazia fasciata* (Curran, 1932)	Tungurahua	Juive (1900)	Fluke 1942: 13
	Tungurahua	Baños (1900)	Fluke 1942: 13
	Imbabura	Cuicocha (3200)	Fluke 1942: 13
	Ecuador		Fluke 1950a: 146
*Fazia fascifrons* (Macquart, 1846)	Bolívar	Hda. Talahua (3100)	Fluke 1942: 12 (as *Epistrophe armillata*)
	Ecuador		Fluke 1950a: 145 (as *Epistrophe armillatus*)
*Fazia imitator* (Curran, 1925)	Tungurahua	Río Mapoto (1400)	Fluke 1942: 11
*Fazia luna* (Fluke, 1942)	Tungurahua	Volcán Tungurahua, Minza Chica (3200)	Fluke 1942: 8
	Bolívar	Hda. Talahua (3100)	Fluke 1942: 8
	Ecuador		Fluke 1950a: 146
*Fazia micrura* (Osten Sacken, 1877)	Morona Santiago	Sucúa	Fluke 1942: 14
	Morona Santiago	Macas	Fluke 1942: 14
	Carchi	Tulcán	Campos 1960: 26 (as *Sphaerophoria picticauda*)
*Fazia remigis* (Fluke, 1942)	Tungurahua	Volcán Tungurahua, Minza Chica (3200)	Fluke 1942: 9
	Bolívar	Hda. Talahua (3100)	Fluke 1942: 9
	Ecuador		Fluke 1950a: 145
*Fazia roburoris* (Fluke, 1942)	Bolívar	Hda. Talahua (3100)	Fluke 1942: 11
	Ecuador		Fluke 1950a: 146
*Hypselosyrphus marshalli* Reemer, 2013	Napo	Tiputini Diodiversity Station	Reemer 2013: 28
*Leucopodella boadicea* (Hull, 1943)	El Oro	Piñas (1506)	Hull 1943i: 73
*Leucopodella delicatula* (Hull, 1943)	Tungurahua	Baños	Hull 1943i: 78
*Leucopodella gracilis* (Williston, 1891)	Ecuador		Thompson et al. 1976: 46 (as *L. asthenia*)
*Leucopodella zenilla* (Hull, 1943)	Bolívar	Hda. Talahua (3100)	Hull 1943g: 77
*Lycastrirhyncha nitens* Bigot, 1859	Ecuador		Montoya et al. 2012: supplementary material page 3; Montoya et al. 2016: 492
*Mallota aberrans* Shannon, 1927	Napo	7 km S of Baeza (2000)	Thompson and Zumbado 2002: 93
*Mallota nigra* Shannon, 1927	Pastaza	Santa Inés	Shannon 1927: 17
*Mallota rubicunda* Curran, 1940	Tungurahua	Volcán Tungurahua (2600)	Curran 1940: 13
*Meromacrus laconicus* (Walker, 1852)	Guayas	Isla Puná (253)	Blatch et al. 2003: 26
*Meromacrus panamensis* Curran, 1930	Guayas	San Eduardo	Campos 1960: 29
*Meromacrus pratorum* (Fabricius, 1775)	Ecuador		Thompson et al. 1976: 113
*Meropidia rufa* Thompson, 1983	Morona Santiago	Limón Indanza (900)	Hippa and Thompson 1983: 110
Microdon (Chymophila) fulgens Wiedemann, 1830	Guayas	Guayaquil, San Eduardo	Campos 1960: 24
Microdon (Microdon) violaceus (Macquart, 1842)	Guayas	Durán	Campos 1960: 24
*Microdon* sp.	Guayas	env. of Guayaquil	Campos 1960: 24
*Mixogaster thecla* (Hull, 1954)	Ecuador		Thompson et al. 1976: 59
Ocyptamus (Calostigma) elnora (Shannon, 1927)	Ecuador		Thompson et al. 1976: 17
Ocyptamus (Hermesomyia) wulpianus (Lynch Arribalzaga, 1891)	Pastaza	Puyo (1250)	Hull 1943a: 50 (as Baccha phobifer)
	Pichincha	40 km SW Quito, Tandapi (1300–1500)	Vockeroth 1969: 123 (as *Hermesomyia bacchiformis*)
Ocyptamus (Hybobathus) flavipennis (Wiedemann, 1830)	Ecuador		Thompson et al. 1976: 18
Ocyptamus (Mimocalla) bonariensis (Curran, 1941)	Tungurahua	Baños	Curran 1941: 284 (as *Salpingogaster flukei*)
	Tungurahua	Baños, Chaupi	Hull 1943a: 51 (as Baccha phobia)
Ocyptamus (Ocyptamus) aeolus (Hull, 1943)	Pastaza	Machai, Río Pastaza (1300)	Hull 1943g: 70
Ocyptamus (Ocyptamus) anonus (Hull, 1943)	Pastaza	Puyo (1000)	Hull 1943d: 91
Ocyptamus (Ocyptamus) cultratus (Austen, 1893)	Manabí	Palmar	Hull 1943g: 78 (as *Baccha satyra*)
Ocyptamus (Ocyptamus) cymbellina (Hull, 1944)	Santo Domingo de los Tsáchilas	Santo Domingo (950)	Hull 1944b: 64
Ocyptamus (Ocyptamus) dimidiatus (Fabricius, 1781)	Guayas	Guayaquil, San Eduardo	Campos 1960: 24
	Guayas	San Eduardo	Campos 1960: 24
Ocyptamus (Ocyptamus) niobe (Hull, 1943)	Manabí	Palmar (200)	Hull 1943i: 74
Ocyptamus (Ocyptamus) princeps (Hull, 1944)	Pastaza	Puyo (1000)	Hull 1944b: 57
Ocyptamus (Ocyptamus) saffrona (Hull, 1943)	Manabí	Palmar	Hull 1943i: 74
Ocyptamus (Ocyptamus) zilla (Hull, 1943)	Pastaza	Puyo	Hull 1943j: 215
Ocyptamus (Orphnabaccha) cerberus (Hull, 1943)	Imbabura	Cuicocha	Hull 1943g: 67
Ocyptamus (Orphnabaccha) opacus (Fluke, 1950)	Tungurahua	Volcán Tungurahua (2800)	Fluke 1950b: 444
	Tungurahua	Baños (1900)	Fluke 1950b: 444
	Napo-Orellana	Sumaco [as Río Zumac] (1400)	Fluke 1950b: 444
Ocyptamus (Orphnabaccha) pteronis (Fluke, 1942)	Tungurahua	Volcán Tungurahua, Minza Chica (3200)	Fluke 1942: 5
	Bolívar	Hda. Talahua	Fluke 1942: 5
	Ecuador		Fluke 1950a: 145
Ocyptamus (Orphnabaccha) trabis (Fluke, 1942)	Tungurahua	Volcán Tungurahua, Runtun (2900)	Fluke 1942: 6
	Imbabura	Cuicocha (3200)	Fluke 1942: 6
	Pichincha	Páramo de Pasachoa	Fluke 1942: 6
	Ecuador		Fluke 1950a: 145
Ocyptamus (Orphnabaccha) virga (Fluke, 1942)	Imbabura	Cuicocha (3200)	Fluke 1942: 7
Ocyptamus (Orphnabaccha) volcanus (Fluke, 1942)	Pastaza	Santa Inés, Río Pastaza (1200)	Fluke 1942: 7
Ocyptamus (Pipunculosyrphus) scintillans (Hull, 1943)	Guayas	Morro (1500)	Hull 1943e: 136
Ocyptamus (Styxia) eblis (Hull, 1943)	Bolívar	Hda. Talahua	Hull 1943g: 66
*Ocyptamus* sp.	Guayas	Guayaquil, San Eduardo	Campos 1960: 24
*Ornidia major* Curran, 1930	Ecuador		Thompson et al. 1976: 69; Thompson 1991: 255
*Ornidia obesa* (Fabricius, 1775)	Galápagos		Peck 1996
	Galápagos	Isabela	Peck et al. 1998: 228; Causton et al. 2006: 135; Sinclair 2015; Sinclair et al. 2016: 84
	Galápagos	San Cristóbal	Peck et al. 1998: 228; Causton et al. 2006: 135; Sinclair 2015; Sinclair et al. 2016: 84
	Galápagos	Santa Cruz	Sinclair 2015
	Ecuador		Thompson et al. 1976: 69; Thompson 1991: 257
	Guayas	Guayaquil	Campos 1960: 26
	Guayas*	El Salado	Campos 1960: 26
	Guayas	San Eduardo	Campos 1960: 26
	Guayas	Durán	Campos 1960: 26
	Guayas	Naranjito	Campos 1960: 26
	Guayas	San Rafael	Campos 1960: 26
	Guayas	Barraganetal	Campos 1960: 26
	Guayas	Bucay	Campos 1960: 26
	Guayas	Posorja	Campos 1960: 26
	Guayas	Playas del Morro	Campos 1960: 26
	Guayas	Naranjal	Campos 1960: 26
	Zamora Chinchipe	Valle del Zamora	Campos 1960: 26
*Ornidia obesa* (Fabricius, 1775)	Loja	Loja	Campos 1960: 26
	Esmeraldas	Telembí, Río Cayapas	Campos 1960: 27
*Palpada aemula* (Williston, 1891)	Ecuador		Montoya et al. 2012: supporting information, page 5; Montoya et al. 2016: 498
*Palpada albifrons* (Wiedemann, 1830)	Galápagos	Santa Cruz	Sinclair 2015
	Galápagos	Floreana	Sinclair et al. 2016: 81
	Galápagos	Isabela	Sinclair et al. 2016: 81
	Galápagos	Marchena	Sinclair et al. 2016: 82
	Guayas	San Eduardo	Campos 1960: 28 (as *Eristalis albiceps*)
*Palpada atrimana* (Loew, 1866)	Ecuador		Montoya et al. 2016: 496
*Palpada conica* (Fabricius, 1805)	Napo	Tena	Morales and Marinoni 2009: 320
	Zamora Chinchipe		Morales and Marinoni 2009: 320
*Palpada cosmia* (Schiner, 1868)	Ecuador		Thompson et al. 1976: 104
*Palpada erratica* (Curran, 1930)	Ecuador		Thompson et al. 1976: 105
	Azuay		Morales and Marinoni 2009: 332
	Sucumbíos		Morales and Marinoni 2009: 332
*Palpada fasciata* (Wiedemann, 1819)	Ecuador		Thompson et al. 1976: 105
	Guayas	Guayaquil	Campos 1960: 28
	Guayas	San Eduardo	Campos 1960: 28
*Palpada funerea* (Rondani, 1851)	Ecuador	Río Napo	Rondani 1851: 357
*Palpada furcata* Wiedemann, 1819)	Pichincha	Quito	Macquart 1855: 110 (as *Eristalis quitensis*)
	Ecuador		Thompson et al. 1976: 106
*Palpada geniculata* (Fabricius, 1805)	Guayas	Guayaquil	Campos 1960: 28 (as *Eristalis obsoletus*)
*Palpada macula* (Sack, 1941)	Ecuador		Thompson et al. 1976: 106
*Palpada mexicana* (Macquart, 1847)	Ecuador		Thompson et al. 1976: 110 (as *Palpada testaceicornis*)
*Palpada monticola* (Röder, 1892)	Carchi	10 km SW Tulcan (2900)	Thompson 1997: 232 (as *Palpada eristaloides*)
	Carchi	Troya (2950)	Thompson 1997: 232 (as *Palpada eristaloides*)
	Azuay	Cerro Tinajillas (3100)	Thompson 1997: 232 (as *Palpada eristaloides*)
	Napo	0°22’S 78°8’W (3500)	Thompson 1997: 232 (as *Palpada eristaloides*)
*Palpada pusilla* (Macquart, 1842)	Ecuador		Thompson et al. 1976: 108
	Guayas	Durán	Campos 1960: 28 (as *Eristalis tricolor*)
*Palpada pusio* (Wiedemann, 1830)	Ecuador		Thompson et al. 1976: 108
*Palpada ruficeps* (Macquart, 1842)	Ecuador		Thompson et al. 1976: 108
*Palpada rufiventris* (Macquart, 1846)	Ecuador		Thompson et al. 1976: 108
*Palpada scutellaris* (Fabricius, 1805)	Napo		Morales and Marinoni 2009: 344
	Pastaza		Morales and Marinoni 2009: 344
	Guayas	Guayaquil	Campos 1960: 28
	Guayas*	El Salado	Campos 1960: 28
	Guayas	San Eduardo	Campos 1960: 28
	Guayas	Durán	Campos 1960: 28
	Guayas	Yaguachi	Campos 1960: 28
	Guayas	Naranjito	Campos 1960: 28
	Guayas	San Rafael	Campos 1960: 28
*Palpada scutellaris* (Fabricius, 1805)	Guayas	Bucay	Campos 1960: 28
	Guayas	Posorja	Campos 1960: 28
	Guayas	Isla Puná, Puerto Grande	Campos 1960: 28
*Palpada suprarufa* Thompson, 1999	Imbabura	S Otavalo (3100–3300)	Thompson 1999: 345
	Napo	Papallacta (2900)	Thompson 1999: 345
	Pichincha	28 miles S Quito	Thompson 1999: 345
	Cañar	Pimo (3200)	Thompson 1999: 345
*Palpada urotaenia* (Curran, 1930)	Ecuador		Thompson et al. 1976: 110
*Palpada vinetorum* (Fabricius, 1799)	Galápagos	Española	Sinclair et al. 2016: 82
	Galápagos	San Cristóbal	Sinclair et al. 2016: 82
	Galápagos	Santa Cruz	Linsley 1977: 39; Sinclair 2015; Sinclair et al. 2016: 82
	Ecuador		Thompson et al. 1976: 110
	Guayas	Guayaquil	Campos 1960: 28
	Guayas	San Eduardo	Campos 1960: 28
*Pelecinobaccha adspersa* (Fabricius, 1805)	Napo	Jatun Sacha Biol. Res. 6 km E Misahuali (450)	Miranda et al. 2014: 18
	Ecuador		Thompson et al. 1976: 12
*Pelecinobaccha andrettae* Miranda, 2014	Napo	Jatun Sacha Biol. Res. 6 km E Misahuali (450)	Miranda et al. 2014: 22
	Pastaza	Pompeya, Napo R.	Miranda et al. 2014: 24
*Pelecinobaccha avispas* Miranda, 2014	Napo	Coca, Napo R. (250)	Miranda et al. 2014: 26
*Pelecinobaccha brevipennis* (Schiner, 1868)	Napo	Coca, Napo R. (250)	Miranda et al. 2014: 30
*Pelecinobaccha clarapex* (Wiedemann, 1830)	Pichincha	Río Palenque Station (250)	Miranda et al. 2014: 33
*Pelecinobaccha dracula* (Hull, 1943)	El Oro	Piñas (1200)	Hull 1943j: 215 (as *Baccha nerissa*); Hull 1949: 162 (as *Baccha nerissa*)
*Pelecinobaccha ida* (Curran, 1941)	Napo	7 km S Baeza	Miranda et al. 2014: 49
*Pelecinobaccha ovipositoria* (Hull, 1943)	Napo	Jatun Sacha Biol. Res. 6 km E Misahuali (450)	Miranda et al. 2014: 62
*Pelecinobaccha pilipes* (Schiner, 1868)	Sucumbíos	Limoncocha (250)	Miranda et al. 2014: 67
	Napo	Coca, Napo R. (250)	Miranda et al. 2014: 67
*Pelecinobaccha transatlantica* (Schiner, 1868)	Napo	Lago Agrio, 41 km W	Miranda et al. 2014: 78
	Orellana	Yasuni Research Stn. (250)	Miranda et al. 2014: 78
	Pastaza	Santa Clara	Miranda et al. 2014: 78
	Sucumbíos	Limoncocha (250)	Miranda et al. 2014: 78
	Zamora Chinchipe	Cumbaratza (700)	Miranda et al. 2014: 78
	Napo	Puerto Misahuallí (350)	Miranda et al. 2014: 78
	Pastaza	Pompeya, Napo R.	Miranda et al. 2014: 78
*Peradon aureus* (Hull, 1944)	Napo	Jatun Yacu, Río Naxo, Watershed (700)	Hull 1944a: 36
Platycheirus (Carposcalis) chalconotus (Philippi, 1865)	Chimborazo	Ríobamba (2700)	Fluke 1945: 16
	Azuay	Cuenca (2500)	Fluke 1945: 16
Platycheirus (Carposcalis) ecuadoriensis (Fluke, 1945)	Imbabura	Cuicocha (3200)	Fluke 1945: 16
	Azuay	Cuenca (2500)	Fluke 1945: 16
	Bolívar	Hda. Talahua (3100)	Fluke 1945: 16
	Chimborazo	Ríobamba (2700)	Fluke 1945: 16
	Chimborazo	Ríobamba (2800)	Fluke 1945: 16
	Pichincha	Uyumbicho (2650)	Fluke 1945: 16
	Pichincha	Chillo Valley, Hda. Teno (2500)	Fluke 1945: 16
	Ecuador		Fluke 1958: 265
Platycheirus (Carposcalis) inflatifrons (Fluke, 1945)	Bolívar	Hda. Talahua (3100)	Fluke 1945: 21
	Ecuador		Fluke 1958: 265
Platycheirus (Carposcalis) punctulatus (Wulp, 1888)	Ecuador	(2100–3300)	Fluke 1945: 15
Platycheirus (Carposcalis) cf. saltanus (Enderlein, 1938)	Ecuador	(4200)	Fluke 1945: 15
Platycheirus (Carposcalis) scutigera (Fluke, 1945)	Pichincha	Uyumbicho (2700)	Fluke 1945: 20
Platycheirus (Carposcalis) stegnus (Say, 1829)	Santa Elena	La Rinconada	Campos 1960: 24
	Chimborazo	Alausí	Campos 1960: 24
	Carchi	El Ángel	Campos 1960: 24
	Pichincha	Casitagua	Campos 1960: 24
	Carchi	Tulcán	Campos 1960: 24
Pseudodoros (Dioprosopa) clavatus (Fabricius, 1794)	Galápagos	Baltra	Sinclair et al. 2016: 89
	Galápagos	Española	Kassebeer 2000: 83; Sinclair et al. 2016: 89
	Galápagos	Genovesa	Kassebeer 2000: 83; Sinclair et al. 2016: 89
	Galápagos	Floreana	Smith 1877: 84 (as *Syrphus albomaculatus*); Coquillett 1901: 374; Linsley and Usinger 1966: 168; Linsley 1977: 39; Kassebeer 2000: 83; Sinclair 2015; Sinclair et al. 2016: 89
	Galápagos	Isabela	Curran 1934: 154; Linsley and Usinger 1966: 168; Linsley 1977: 39; Kassebeer 2000: 83; Sinclair 2015; Sinclair et al. 2016: 89
	Galápagos	Pinta	Sinclair et al. 2016: 89
	Galápagos	Marchena	Linsley 1977: 39; Sinclair 2015; Sinclair et al. 2016: 89
	Galápagos	San Cristóbal	Curran 1934: 154; Linsley and Usinger 1966: 168; Linsley 1977: 39; Sinclair 2015; Sinclair et al. 2016: 89
	Galápagos	Rábida	Sinclair et al. 2016: 89
	Galápagos	Santiago	Coquillett 1901: 374; Linsley and Usinger 1966: 168; Linsley 1977: 39; Kassebeer 2000: 83
	Galápagos	Santa Fé	Sinclair et al. 2016: 89
	Galápagos	Bartolomé	Kassebeer 2000: 83
	Galápagos	Seymour Norte	Johnson 1924: 88
	Galápagos	Santa Cruz	Linsley 1977: 39; Kassebeer 2000: 83; Sinclair 2015; Sinclair et al. 2016: 89
	Galápagos		Thomson 1869 : 548 (as *Baccha facialis*)
Pseudodoros (Dioprosopa) clavatus (Fabricius, 1794)	Azuay	32 km W Santa Isabel (900)	Kassebeer 2000: 85
	Manabí	Manta-Jipijapa rd. (150)	Kassebeer 2000: 85
	Zamora Chinchipe	Zamora (1500)	Kassebeer 2000: 85
	Zamora Chinchipe	Loja, San Pedro (1550)	Kassebeer 2000: 85
Pseudodoros (Dioprosopa) vockerothi (Kassebeer, 2000)	Bolívar	Chota River, Carchi (1800)	Kassebeer 2000: 76
	Imbabura	Ibarra, Yaguarcocha (2300)	Kassebeer 2000: 76
	Loja	S. Pedro-Zaruma rd Loja (850–1100)	Kassebeer 2000: 76
	Imbabura	Taguando R., NW Ibarra (1650–1900)	Kassebeer 2000: 76
Quichuana aff. quixotea Hull, 1946	Napo	Limoncocha	Ricarte et al. 2012: 129
*Relictanum crassum* (Walker, 1852)	Cotopaxi	Latacunga (330)	Miranda et al. 2014: 91
	Los Ríos	Río Palenque (150)	Miranda et al. 2014: 91
	Napo	Puerto Misahuallí (350)	Miranda et al. 2014: 91
	Sucumbíos	Limoncocha (250)	Miranda et al. 2014: 91
*Relictanum johnsoni* (Curran, 1934)	Napo	Coca, Napo R. (250)	Miranda et al. 2014: 93
Rhingia (Rhingia) longirostris Fluke, 1943	Bolívar	Hda. Talahua (3100)	Fluke 1943: 431
Rhingia (Rhingia) nigra Macquart, 1846	Ecuador		Montoya et al. 2016: 506
*Rhinoprosopa lucifer* (Hull, 1943)	El Oro	Piñas (1600)	Hull 1943j: 216
*Rhinoprosopa nasuta* (Bigot, 1884)	Carchi	R. Chota (2000)	Mengual 2015: 16
*Rhopalosyrphus ecuadoriensis* Reemer, 2013	Orellana	Yasuni Research Station	Reemer and Ståhls 2013a: 119
*Salpingogaster browni* Curran, 1941	Tungurahua	Volcán Tungurahua, Minza Chica (3200)	Curran 1941: 286
*Scaeva melanostoma* (Macquart, 1842)	Azuay		Thompson et al. 1976: 9
	Pichincha	2 km W Cayambe (2300)	Kassebeer 1999: 99
	Carchi	El Ángel (2700)	Kassebeer 1999: 99
	Pichincha	Valle de Machachi (2900)	Kassebeer 1999: 99
	Chimborazo	Riobamba	Campos 1960: 29; Kassebeer 1999: 99
	Chimborazo	env. of Riobamba	Kassebeer 1999: 99
*Scaeva occidentalis* Shannon, 1927	Pichincha	Valle de Machachi (2900)	Kassebeer 1999: 101
Sterphus (Crepidomyia) chloropyga (Schiner, 1868)	Ecuador		Schiner 1868: 366 (type-locality as “Colombien”, referring to Colombia, Ecuador, or Venezeula); Montoya et al. 2016: 504
Sterphus (Crepidomyia) plagiatus (Wiedemann, 1830)	Napo	Napo River, Coca (250)	Thompson 1973: 220
	Napo	Napo River	Thompson 1973: 220
	Pastaza	Napo River, Pompeya	Thompson 1973: 220
Sterphus (Telus) telus Thompson, 1973	Azuay	Tarqui (2800)	Thompson 1973: 198
*Stipomorpha guianica* (Curran, 1925)	Morona Santiago	Limón Indanza (900)	Reemer 2013: 54
	Ecuador		Thompson et al. 1976: 62
*Stipomorpha tenuicauda* (Curran, 1925)	Napo	Jatun Sacha Res., 6 km E Misahualli (450)	Reemer 2013: 70
*Stipomorpha zophera* Reemer, 2013	Napo	Limoncocha	Reemer 2013: 75
Syrphus aff. lacyorum Thompson, 2000	Morona Santiago	Río Blanco	Thompson et al. 2000: 39
*Syrphus reedi* Shannon, 1927	Zamora Chinchipe	Valle de Zamora	Campos 1960: 25
*Syrphus shorae* Fluke, 1950	Tungurahua	Baños (1500–2100)	Fluke 1942: 3 (as *S. willistoni*)
	Tungurahua	Juive (1950)	Fluke 1942: 3 (as *S. willistoni*)
	Pichincha	Hda. San Rafael, Río San Pedro (2700)	Fluke 1942: 3 (as *S. willistoni*)
	Ecuador		Fluke 1950a: 143 (as *S. willistoni*)
*Talahua fervida* (Fluke, 1945)	Bolívar	Hda. Talahua (3100)	Fluke 1945: 23
	Ecuador		Fluke 1958: 266
*Toxomerus anthrax* (Schiner, 1868)	Ecuador		Thompson et al. 1976: 48; Mengual 2011: 9
	Pastaza	Abitagua Oriente	Gerdes 1974a: 14-15
	Tungurahua	Baños	Gerdes 1974a: 14-15
	Pastaza	Cerro Obitahua	Gerdes 1974a: 14-15
	Ecuador**	Conquista	Gerdes 1974a: 14-15
	Tungurahua	Naguazo	Gerdes 1974a: 14-15
	Napo	Napo Oriente	Gerdes 1974a: 14-15
	Pastaza	Obitahua Oriente	Gerdes 1974a: 14-15
	Morona Santiago	Río Blanco	Gerdes 1974a: 14-15
	Morona Santiago	Río Negro	Gerdes 1974a: 14-15
	Tungurahua	Runtun	Gerdes 1974a: 14-15
	Chimborazo	Sangay Oriente	Gerdes 1974a: 14-15
	Pastaza	Puerto Santana	Gerdes 1974a: 14-15
	Pastaza	Sarayacu	Gerdes 1974a: 14-15
	Pastaza	Sarayacu Oriente	Gerdes 1974a: 14-15
	Pastaza	El Topo	Gerdes 1974a: 14-15
	Pichincha	Chaupi	Gerdes 1974a: 14-15
	Tungurahua	Ulvilla	Gerdes 1974a: 14-15
	Chimborazo	Chilicay	Mengual 2011: appendix 1
	Chimborazo	Huigra	Mengual 2011: appendix 1
	El Oro	Portovelo	Mengual 2011: appendix 1
*Toxomerus antiopa* (Hull, 1951)	Bolívar	Hda. Talahua (3100)	Hull 1951: 5
	Chimborazo	Urbina (3650)	Hull 1951: 5
*Toxomerus aquilinus* Sack, 1941	Ecuador		Metz and Thompson 2001: 233
*Toxomerus arcifer* (Loew, 1866)	Ecuador		Thompson et al. 1976: 48
*Toxomerus brevifacies* (Hull, 1943)	Tungurahua	Baños, Runtun trail	Hull 1943g: 20
	Imbabura	Cuicocha	Hull 1943g: 20
	Pastaza	San Francisco	Hull 1943g: 20
	Tungurahua	Juive	Hull 1943g: 20
	Tungurahua	Baños	Hull 1943g: 20; Gerdes 1974a: 19
	Azuay	Cuenca	Hull 1943g: 20
	Tungurahua	Baños	Gerdes 1974a: 19; Gerdes 1975: 20
	Pichincha	Chaupi	Gerdes 1974a: 20; Gerdes 1975: 20
	Ecuador**	Conquista	Gerdes 1974a: 20; Gerdes 1975: 20
	Pastaza	Obitagua	Gerdes 1974a: 20; Gerdes 1975: 20
	Morona Santiago	Río Blanco	Gerdes 1974a: 20; Gerdes 1975: 20
	Morona Santiago	Río Negro	Gerdes 1974a: 20; Gerdes 1975: 20
	Tungurahua	Runtun	Gerdes 1974a: 20; Gerdes 1975: 20
	Chimborazo	Sangay Oriente	Gerdes 1974a: 20; Gerdes 1975: 20
	Pastaza	Sarayacu	Gerdes 1974a: 20; Gerdes 1975: 20
	Pastaza	Sarayacu Oriente	Gerdes 1974a: 20; Gerdes 1975: 20
	Pastaza	Topo	Gerdes 1974a: 20; Gerdes 1975: 20
	Tungurahua	Ulvilla	Gerdes 1974a: 20; Gerdes 1975: 20
	Pastaza	Abitagua Oriente	Gerdes 1974a: 20; Gerdes 1975: 20
	Tungurahua	Naguazo	Gerdes 1974a: 20; Gerdes 1975: 20
	Pastaza	Puerto Santana	Gerdes 1974a: 20; Gerdes 1975: 20
*Toxomerus claracuneus* (Hull, 1942)	Pastaza	Río Margaritas, Río Pastaza (1250)	Hull 1942: 107
	Ecuador**	Conquista	Gerdes 1974a: 22
	Pastaza	Puerto Santana	Gerdes 1974a: 22
*Toxomerus crockeri* (Curran, 1934)	Galápagos	Floreana	Curran 1934: 155; Linsley and Usinger 1966: 168; Linsley 1977: 39; Sinclair and Peck 2002; Sinclair 2015; Sinclair et al. 2016: 91
	Galápagos	Isabela	Curran 1934: 155; Linsley and Usinger 1966: 168; Linsley 1977: 39; Peck 1994; Sinclair and Peck 2002; Boada 2005: 80; Sinclair 2015; Sinclair et al. 2016: 91
	Galápagos	Pinta	Sinclair and Peck 2002
	Galápagos	San Cristóbal	Curran 1934: 155; Linsley and Usinger 1966: 168; Linsley 1977: 39; Sinclair and Peck 2002; Sinclair et al. 2016: 91
	Galápagos	Santiago	Coquillett 1901: 374 (as Mesogramma duplicata); Curran 1934: 155; Linsley and Usinger 1966: 168; Linsley 1977: 39; Sinclair and Peck 2002
	Galápagos	Española	Sinclair et al. 2016: 91
	Galápagos	Pinta	Sinclair et al. 2016: 91
	Galápagos	Santa Cruz	Curran 1934: 155; Boada 2005: 85; Sinclair 2015; Sinclair et al. 2016: 91
*Toxomerus dispar* (Fabricius, 1794)	Tungurahua	Baños	Hull 1943f: 26 (as *Mesogramma basilaris* var. bifida); Gerdes 1974a: 17
	Napo	Napo Oriente	Gerdes 1974a: 17 (as *Toxomerus basilaris*)
	Morona Santiago	Río Blanco	Gerdes 1974a: 17 (as *Toxomerus basilaris*)
	Ecuador		Mengual 2011: 13
*Toxomerus duplicatus* (Wiedemann, 1830)	Pichincha	Pichincha	Hull 1943f: 18 (as *Mesogramma arcturus*)
	Pichincha	Tío Loma	Campos 1960: 25
	Napo	Napo Oriente	Gerdes 1974a: 23
*Toxomerus ecuadoreus* (Hull, 1943)	Azuay	Cuenca (2500)	Hull 1943g: 20
	Tungurahua	Baños (2200)	Hull 1943g: 20
	Pichincha	Pichincha (2500)	Hull 1943g: 20
	Pichincha	Hda. San Rafael, Río San Pedro	Hull 1943g: 20; Gerdes 1974a: 26
	Pichincha	Uyumbicho	Hull 1943g: 20; Gerdes 1974a: 26
	Tungurahua	Baños, Río Pablo (2200)	Hull 1943g: 20
	Tungurahua	Baños, Runtun	Hull 1943g: 20
	Chimborazo	Ríobamba (2700)	Hull 1943g: 20
	Pichincha	Aloag	Gerdes 1974a: 26; Gerdes 1975: 22
	Tungurahua	Baños	Gerdes 1975: 22
	Pastaza	Obitagua	Gerdes 1974a: 26; Gerdes 1975: 22
	Morona Santiago	Río Blanco	Gerdes 1974a: 26; Gerdes 1975: 22
	Pichincha	Chaupi	Gerdes 1974a: 26
	Tungurahua	Ulvilla	Gerdes 1974a: 26
	Morona Santiago	Río Negro	Gerdes 1974a: 26; Gerdes 1975: 22
	Tungurahua	Runtun	Gerdes 1974a: 26; Gerdes 1975: 22
*Toxomerus flaviplurus* (Hall, 1927)	Pastaza	Cerro Obitahua	Gerdes 1974a: 31
	Napo	Napo Oriente	Gerdes 1974a: 31
	Pastaza	Puyo Oriente	Gerdes 1974a: 31
	Chimborazo	Sangay Oriente	Gerdes 1974a: 31
	Pastaza	1.5 km S Puyo, Río Pido Grande	Mengual 2011: appendix 1
	Tungurahua	32 km E Baños (1560)	Mengual 2011: appendix 1
	Napo	Tena	Mengual 2011: appendix 1
	Napo	Santa Cecilia	Mengual 2011: appendix 1
	Napo	60 km W LagoAgRío	Mengual 2011: appendix 1
	Napo	Limoncocha	Mengual 2011: appendix 1
	Zamora Chinchipe	Zumbi	Mengual 2011: appendix 1
	Zamora Chinchipe	Cumbaratza	Mengual 2011: appendix 1
	Zamora Chinchipe	Yantzaza	Mengual 2011: appendix 1
*Toxomerus floralis* (Fabricius, 1789)	Ecuador		Thompson and Thompson 2007: 324
	Napo	Napo Oriente	Gerdes 1974a: 35
*Toxomerus hieroglyphicus* (Schiner, 1868)	Tungurahua	Baños	Gerdes 1974a: 37; Mengual 2011: appendix 1
	Ecuador		Thompson et al. 1976: 51; Mengual 2011: 16
	Pastaza	Cerro Obitahua	Gerdes 1974a: 37
	Pastaza	Obitahua Oriente	Gerdes 1974a: 37
	Morona Santiago	Río Blanco	Gerdes 1974a: 37
	Tungurahua	Runtun	Gerdes 1974a: 38
	Chimborazo	Sangay Oriente	Gerdes 1974a: 38
	Pastaza	Abitagua Oriente	Gerdes 1974a: 38
	Ecuador**	Conquista	Gerdes 1974a: 38
*Toxomerus idalius* (Hull, 1951)	Pastaza	Puyo (1000)	Hull 1951: 12; Hull 1951: 13 (as *Mesogramma idalia leda*)
	Pastaza	Río Pastaza, San Francisco (1200)	Hull 1951: 13 (as *Mesogramma idalia leda*); Hull 1951: 18 (as *Mesogramma eurydice*)
*Toxomerus insignis* (Schiner, 1868)	Ecuador		Thompson et al. 1976: 50 (as *T. elongatus*); Metz and Thompson 2001: 235
	Tungurahua	Baños	Gerdes 1974a: 29 (as *Toxomerus elongatus*)
	Napo	Napo Oriente	Gerdes 1974a: 29 (as *Toxomerus elongatus*)
	Tungurahua	Ulvilla	Gerdes 1974a: 29 (as *Toxomerus elongatus*)
	Pastaza	Abitagua	Gerdes 1974a: 29 (as *Toxomerus elongatus*)
	Pastaza	Sarayacu	Gerdes 1974a: 29 (as *Toxomerus elongatus*)
	Pastaza	Abitagua	Gerdes 1974a: 29 (as *Toxomerus elongatus*)
*Toxomerus lacrymosus* (Bigot, 1884)	Napo	Napo Oriente	Gerdes 1974a: 40
	Pastaza	Obitahua Oriente	Gerdes 1974a: 40
	Nariño [Colombia]**	Piedrancha	Gerdes 1974a: 40
	Chimborazo	Sanqay Oriente	Gerdes 1974a: 40
	Pastaza	Sarayacu	Gerdes 1974a: 40
*Toxomerus laenas* (Walker, 1852)	Ecuador		Thompson et al. 1976: 53 (as *T. nitidiventris*)
*Toxomerus marginatus* (Say, 1823)	Cañar-Chimborazo	Quinua-Loma	Campos 1960: 25
*Toxomerus minutus* (Wiedemann, 1830)	Pichincha	Casitagua	Campos 1960: 26
	Carchi	El Vínculo	Campos 1960: 26
	Azuay	Borma	Campos 1960: 26
	Santa Elena	La Rinconada	Campos 1960: 26
	Cañar-Chimborazo	Quinua-Loma	Campos 1960: 26
	Santo Domingo de los Tsáchilas	Santo Domingo de los Colorados	Campos 1960: 26
	Carchi	Tulcán	Campos 1960: 26
	Loja	Loja	Campos 1960: 26
*Toxomerus nasutus* Sack, 1941	Pichincha	Uyumbicho (2700)	Hull 1951: 8 (as *Mesogramma ultima*)
	Tungurahua	Baños (2500)	Hull 1943c: 36 (as *Mesogramma sylpha*)
	Tungurahua	Baños (1800)	Hull 1943c: 36 (as *Mesogramma sylpha*)
	Tungurahua	Baños	Gerdes 1975: 14
	Pichincha	Chaupi	Gerdes 1974a: 42; Gerdes 1975: 14
	Ecuador**	Conquista	Gerdes 1974a: 42; Gerdes 1975: 14
	Tungurahua	Naguazo	Gerdes 1974a: 42; Gerdes 1975: 14
	Napo	Napo Oriente	Gerdes 1974a: 42; Gerdes 1975: 14
*Toxomerus nasutus* Sack, 1941	Pastaza	Obitagua	Gerdes 1974a: 42; Gerdes 1975: 14
	Pastaza	Obitahua	Gerdes 1974a: 42; Gerdes 1975: 14
	Pastaza	Abitagua Oriente	Gerdes 1974a: 42
	Pastaza	Cerro Obitahua	Gerdes 1974a: 42
	Manabí*	San José	Gerdes 1974a: 42
	Tungurahua	El Topo	Gerdes 1974a: 43
	Morona Santiago	Río Blanco	Gerdes 1974a: 42; Gerdes 1975: 14
	Morona Santiago	Río Negro	Gerdes 1974a: 42; Gerdes 1975: 14
	Tungurahua	Runtun	Gerdes 1974a: 42; Gerdes 1975: 14
	Tungurahua*	El Salado	Gerdes 1974a: 42; Gerdes 1975: 14
	Chimborazo	Sangay	Gerdes 1974a: 43; Gerdes 1975: 14
	Pastaza	Puerto Santana	Gerdes 1974a: 43; Gerdes 1975: 14
	Pastaza	Sarayacu	Gerdes 1974a: 43; Gerdes 1975: 14
	Pastaza	Sarayacu Oriente	Gerdes 1974a: 43; Gerdes 1975: 14
	Pichincha*	Yunguilla	Gerdes 1974a: 44; Gerdes 1975: 14
*Toxomerus norma* (Hull, 1941)	Ecuador		Thompson et al. 1976: 52 (as *T. mulio*); Metz and Thompson 2001: 239 (as *T. mulio*)
*Toxomerus nymphalius* (Hull, 1942)	Pastaza	Río Margaritas (1250)	Hull 1942: 106
	Morona Santiago	Sucúa, Río Blanco (950)	Hull 1942: 106
	Pastaza	Puyo	Hull 1942: 106
	Pastaza	Río Mapeto	Hull 1942: 106
	Pastaza	Cerro Obitahua	Gerdes 1974a: 46
	Pastaza	Obitahua Oriente	Gerdes 1974a: 46
	Chimborazo	Sangay Oriente	Gerdes 1974a: 46
	Pastaza	Sasayacu Oriente	Gerdes 1974a: 46
	Pichincha*	Yunguilla	Gerdes 1974a: 46
*Toxomerus parvulus* (Loew, 1866)	Ecuador		Thompson et al. 1976: 55 (as *T. slossonae*)
*Toxomerus pichinchae* Gerdes, 1974	Pichincha	Aloag (2600)	Gerdes 1974b: 280
*Toxomerus pictus* (Macquart, 1842)	Pastaza	Cerro Obitahua	Gerdes 1974a: 48
	Chimborazo	Sangay Oriente	Gerdes 1974a: 48
	Napo	Napo Oriente	Gerdes 1974a: 49
*Toxomerus picudus* Mengual, 2011	Orellana	Estación Tiputini (227)	Mengual 2011: 21
*Toxomerus politus* (Say, 1823)	Galápagos	Floreana	Sinclair 2015
	Galápagos	Isabela	Sinclair 2015
	Galápagos	Santa Cruz	Sinclair et al. 2016: 93
	Galápagos	San Cristóbal	Sinclair 2015
	Galápagos	Santiago	Sinclair et al. 2016: 93
	Tungurahua	Baños	Gerdes 1974a: 51
	Napo	Napo Oriente	Gerdes 1974a: 51
	Nariño [Colombia]**	Piedrancha	Gerdes 1974a: 51
	Pastaza	Sarayacu	Gerdes 1974a: 51
	Ecuador		Thompson et al. 1976: 53; Metz and Thompson 2001: 241
*Toxomerus porticola* (Thomson, 1869)	Ecuador		Thompson et al. 1976: 54
*Toxomerus productus* (Curran, 1930)	Morona Santiago	Macas, Río Upano (1000)	Hull 1951: 10 (as *Mesogramma cyrilla*)
	Ecuador		Curran 1930: 5
	Napo	Napo Oriente	Gerdes 1974a: 53; Gerdes 1975: 16
	Pastaza	Obitahua	Gerdes 1974a: 53; Gerdes 1975: 16
	Chimborazo	Sangay	Gerdes 1974a: 53; Gerdes 1975: 16
	Pastaza	Sarayacu	Gerdes 1974a: 53; Gerdes 1975: 16
	Pastaza	Sarayacu	Gerdes 1974a: 54; Gerdes 1975: 16
	Tungurahua	Baños	Gerdes 1974a: 54; Gerdes 1975: 16
	Pastaza	Obitagua	Gerdes 1974a: 54; Gerdes 1975: 16
	Morona Santiago	Río Negro	Gerdes 1974a: 54; Gerdes 1975: 16
	Tungurahua	Runtun	Gerdes 1974a: 54; Gerdes 1975: 16
	Pastaza	Sarayacu Oriente	Gerdes 1974a: 54; Gerdes 1975: 16
*Toxomerus rombicus* (Giglio-Tos, 1892)	Azuay	Cuenca	Campos 1960: 25
*Toxomerus saphiridiceps* (Bigot, 1884)	Ecuador		Thompson et al. 1976: 50 (as *T. flavus*), 54; Metz and Thompson 2001: 246
	Tungurahua	Baños	Gerdes 1974a: 33 (as *Toxomerus flavus*)
	Ecuador**	Conquista	Gerdes 1974a: 34 (as *Toxomerus flavus*)
	Morona Santiago	Río Blanco	Gerdes 1974a: 34 (as *Toxomerus flavus*)
	Manabí*	San José	Gerdes 1974a: 34 (as *Toxomerus flavus*)
	Nariño [Colombia]**	Piedrancha	Gerdes 1974a: 34 (as *Toxomerus flavus*)
	Tungurahua	Runtun	Gerdes 1974a: 34 (as *Toxomerus flavus*)
	Pastaza	Sarayacu	Gerdes 1974a: 34 (as *Toxomerus flavus*)
	Los Ríos	Soledad	Gerdes 1974a: 34 (as *Toxomerus flavus*)
*Toxomerus* sp.	Galápagos	Santa Cruz	Boada 2005: 86
*Toxomerus* sp.	Guayas	San Eduardo	Campos 1960: 26
	Guayas	Guayaquil	Campos 1960: 26
	Guayas	Durán	Campos 1960: 26
*Toxomerus steatogaster* (Hull, 1941)	Morona Santiago	Sucúa, Río Blanco and Río Upano (950)	Hull 1943f: 21 (as *Mesogramma steatornis*)
	Pastaza	Puyo (1000)	Hull 1943f: 21 (as *Mesogramma steatornis*)
	Napo	Napo Oriente	Gerdes 1974a: 55
	Ecuador		Thompson et al. 1976: 55
*Toxomerus sylvaticus* (Hull, 1943)	Tungurahua	Baños	Hull 1943c: 35; Gerdes 1974a: 57
	Pastaza	Cerro Obitahua	Gerdes 1974a: 57
	Pastaza	Obitahua Oriente	Gerdes 1974a: 57
	Morona Santiago	Río Blanco	Gerdes 1974a: 57
	Chimborazo	Sanqay Oriente	Gerdes 1974a: 57
	Pichincha	Chaupi	Gerdes 1974a: 57
*Toxomerus tibicen* (Wiedemann, 1830)	Guayas	Guayaquil, San Eduardo	Campos 1960: 25
*Toxomerus tubularius* (Hull, 1942)	Tungurahua	Baños (2000)	Hull 1942: 104
*Toxomerus virgulatus* (Macquart, 1850)	Ecuador		Thompson et al. 1976: 49 (as *T. confusus*)
*Toxomerus watsoni* (Curran, 1930)	Ecuador		Thompson et al. 1976: 56
*Tuberculanostoma antennatum* Fluke, 1943	Bolívar	Talahua (3100)	Fluke 1943: 426
	Ecuador		Fluke 1958: 266
*Tuberculanostoma browni* Fluke, 1943	Chimborazo	Urbina (3650)	Fluke 1943: 429
	Bolívar	Hda. Talahua (3100)	Fluke 1943: 430
	Bolívar	Cumbre de Tililac (4200)	Fluke 1943: 430
*Tuberculanostoma cilium* Fluke, 1943	Tungurahua	Volcán Tungurahua, Minza Chica (3200)	Fluke 1943: 428
	Bolívar	Hda. Talahua (3100)	Fluke 1943: 428
*Tuberculanostoma pectinis* Fluke, 1943	Bolívar	Hda. Talahua (3100)	Fluke 1943: 430
*Ubristes ictericus* Reemer, 2013	Sucumbíos	Sach Lodge (270)	Reemer 2013: 80
Xanthandrus (Xanthandrus) palliatus (Fluke, 1945)	Bolívar	Hda. Talahua (3100)	Fluke 1945: 22
	Tungurahua	Volcán Tungurahua, Minza Chica (3200)	Fluke 1945: 22

Some original locality names were difficult to place in the current administrative divisions of Ecuador. The Río Pastaza (= Pastaza river) runs through two Ecuadorian provinces, i.e. Pastaza and Morona Santiago, and we used Pastaza province for this locality. On the other hand, Quinua Loma is a locality situated between two provinces, Cañar and Chimborazo, and we listed both provinces in Table 1.

Most of the uncertainties on geographical localities come from Gerdes (1974a). For instance, Gerdes (1974a) named three localities as different ones, i.e. Obitagua, Obitahua, and Abitagua, although we believe that they might refer to the same area. There is a single locality named Abitagua in Ecuador, but instead of assuming all being the same locality, we left the three names in Table 1. We are not sure if the locality San José (Gerdes (1974a) is the one currently situated in Manabí, and there are two localities named El Salado in Guayas (between 0 and 200 masl) and in Tungurahua (circa 2,000 masl). We listed El Salado in Guayas for the records of Campos (1960), as most of the records in that work were from Guayas, but we used Tungurahua for El Salado of Gerdes (1974a, 1975) for the record of *Toxomerus nasutus* Sack, 1941 because other records for this species are close to or over 2,000 masl. We had a similar problem with Yunguilla, a locality also found in two different provinces (Azuay and Pichincha), and we used Pichincha in this case because Gerdes had studied material from Pichincha but not from Azuay. All these records are marked with an asterisk (*) in the Province column of Table 1.

The locality Piedrancha belongs to Colombia (Nariño department), but it was left in Table 1 because Gerdes (1974a) listed it as Ecuador. Finally, we were not able to locate Conquista in Ecuador. These records are marked with two asterisks (**) in the Province column of Table 1.

For the elaboration of Tables 1 and 2, the most recent Syrphidae classification has been used (Mengual et al. 2008, 2009, Thompson 2012, 2013, Reemer and Ståhls 2013a, Miranda et al. 2014, 2016, Mengual 2015). Flower fly species recorded in Ecuador are listed in Table 1 in alphabetical order. Genera with the highest number of species were *Toxomerus* (38), *Ocyptamus* (22) and *Palpada* (21) (Table 2).

**Table 2.** Number of genera and species registered in Ecuador.

Genus	Number of species in Ecuador
*Alipumilio* Shannon, 1927	1
*Allograpta* Osten Sacken, 1875	9
*Argentinomyia* Lynch Arribalzaga, 1891	10
*Claraplumula* Shannon, 1927	1
*Copestylum* Macquart, 1846	19
*Dasysyrphus* Enderlein, 1938	1
*Dolichogyna* Macquart, 1842	2
*Eosalpingogaster* Hull, 1949	1
*Eristalis* Latreille, 1804	1
*Eupeodes* Osten Sacken, 1877	1
*Fazia* Shannon, 1927	12
*Hypselosyrphus* Hull, 1937	1
*Leucopodella* Hull, 1949	4
*Lycastrirhyncha* Bigot, 1859	1
*Mallota* Meigen, 1822	3
*Meromacrus* Rondani, 1848	3
*Meropidia* Hippa & Thompson, 1983	1
*Microdon* Meigen, 1803	3
*Mixogaster* Macquart, 1842	1
*Ocyptamus* Macquart, 1834	22
*Ornidia* Lepeletier & Serville, 1828	2
*Palpada* Macquart, 1834	21
*Pelecinobaccha* Shannon, 1927	10
*Peradon* Reemer, 2013	1
*Platycheirus* Lepeletier & Serville, 1828	7
*Pseudodoros* Becker, 1903	2
*Quichuana* Knab, 1913	1
*Relictanum* Miranda, 2014	2
*Rhingia* Scopoli, 1763	2
*Rhinoprosopa* Hull, 1942	2
*Rhopalosyrphus* Giglio-Tos, 1891	1
*Salpingogaster* Schiner, 1868	1
*Scaeva* Fabricius, 1805	2
*Sterphus* Philippi, 1865	3
*Stipomorpha* Hull, 1945	3
*Syrphus* Fabricius, 1775	3
*Talahua* Fluke, 1945	1
*Toxomerus* Macquart, 1855	38
*Tuberculanostoma* Fluke, 1943	4
*Ubristes* Walker, 1852	1
*Xanthandrus* Verrall, 1901	1

Four unidentified species are listed as such (*Microdon* sp., *Ocyptamus* sp. and two *Toxomerus* sp.), and three species are *affinis* to known species, Dasysyrphus aff. lotus, Syrphus aff. lacyorum and Quichuana aff. quixotea. Ricarte et al. (2012) reviewed the taxonomy of the genus *Quichuana* Knab, 1913 and mentioned one *Quichuana* species recorded for Ecuador (Ricarte et al. 2012: 129, Figure 84). The identity of this species was not stated by Ricarte et al. (2012), but personal communication with A. Ricarte revealed that it is Quichuana aff. quixotea (Hull 1946). Four specimens from Ecuador labelled as *Q. quixotea* are known to be deposited in the USNM collection. However, they show some morphological differences with the holotype that prevented Ricarte et al. (2012) to ascertain their identity (Antonio Ricarte, pers. comm.).

There was some ambiguity with *Peradon oligonax* (Hull, 1944) to either include it or not in the checklist. *Peradon oligonax* was described from Pto. America, Río Putumayo (Hull 1944c). Thompson et al. (1976: 66) indicated the type-locality as part of Ecuador, but Hull (1944c: 36) listed it as Brazil. Putumayo River forms part of Colombia’s border with Ecuador, as well as most of the frontier with Peru, and it ends as a tributary of the Amazon River in Brazil, but there it is known as Içá. Rasmussen (2016) gave details of the Cornell University expedition to South America (collectors of the type material) and he provided evidences that the expedition never went to Ecuador and the expedition was near Javary island (Santo Antônio do Içá) in the dates when the type material was collected. Thus, the type-locality is in Brazil and not in Ecuador, as indicated by Thompson et al. (1976).

Another uncertain taxon was *Priomerus gagathinus* Bigot, 1887, originally described from Loja. Thompson et al (1976) declared the type of this taxon as lost and did not recognize the species. Thompson (2015) indicated that the name *Priomerus* was preoccupied and its species currently belong to four different genera. He did not recognize either the species *gagathinus* Bigot. Thus, we did not list this species in Table 1.

In the literature, we found two doubtful species records, probably due to a misidentification. Sphaerophoria (Sphaerophoria) sulphuripes (Thomson, 1869) is a Nearctic species found along the west coast of the United States and Canada (Knutson 1973). Thompson et al. (1976: 38) listed one specimen identified as *S. sulphuripes* (with no details about the responsible of this identification) in The Natural History Museum (BMNH, London, U.K.) from Ecuador with a question mark. This specimen might be mislabeled or it could be an *Allograpta* specimen, most likely a female, somehow similar to *S. sulphiripes*. We believe that *S. sulphuripes* does not occur in Ecuador and it was not included in Table 1. The other taxon that was misidentified is Eristalis (Eoseristalis) pertinax (Scopoli, 1763), identified by Campos (1960). This species ranges from Fennoscandia south to Iberia and the Mediterranean, and from Ireland through much of Europe into European parts of Russia and Turkey; apparently it is not known beyond the Urals (Speight 2016). We do believe that the record might be an *Eristalis* species, but not *E. pertinax* as it does not occur in the Neotropics. Thus, this record is not listed in Table 1.

Three species are not listed due to the uncertainty of their taxonomic identity. *Syrphus excavatus* (Rondani 1851: 359) and *Syrphus fasciventris* (Rondani 1851: 360), both described from Río Napo, are not included because the type material was not studied and the generic name is probably incorrect. The third species not included is *Xanthandrus* sp. (Curran 1934: 155; from Pinta Island, Galapagos). Sinclair et al. (2016) could not find the material studied by Curran to confirm if the specimen from Galapagos is truly *Xanthandrus* or *Argentinomyia agonis* (Walker 1849).

## Discussion

Montoya et al. (2012) recorded 128 species of 40 different genera for Ecuador, indicating that Ecuador shares a high number of species with Brazil (29 species), Colombia (50) and Peru (29). The present work raises those numbers considerably, up to 201 identified species of 51 genera and subgenera. Based on previous studies, the Ecuadorian diversity of flower flies is comparable to the one from Peru (195 spp., 75 genera; Montoya et al. 2012), Costa Rica (228 species, 41 genera; Montoya et al. 2012) or Suriname (183 species, 36 genera; Reemer 2016). It is important to emphasize that Ecuador is one of the smallest countries in the Neotropics and South America, but it has one of the highest diversity densities for the Neotropics with ca. 7.2 species per 10.000 km2. This diversity density makes Ecuador the third top country after Costa Rica and Suriname, the two most explored and well-studied faunae in the Neotropics. It must also be pointed out that the present work is based only on records from the literature, and authors are sure that the flower fly diversity in Ecuador is higher.

This study confirms the argument of Montoya et al. (2012) when stating that “The understanding of the distribution and composition of Syrphidae in the Neotropical Region remains far from complete”. Since Thompson et al. (1976) there have been mostly taxonomic contributions on the Neotropical flower flies, but little faunistic studies have been published. Thompson (1999) provided a key to the Neotropical genera of Syrphidae, including a glossary of taxonomic terms and the description of a few new species, and Thompson (2006) compiled all the taxonomic knowledge of Neotropical flower flies up to that date, but those cannot be considered faunistic studies. In the *Systema Dipterorum*, Thompson (2013) had some distributional range notes for each species, but the fauna of the Neotropical countries has not been studied more thoroughly yet. The syrphid fauna of three Neotropical countries have been recently revised: a catalogue for Colombia (Montoya 2016, see also Gutierrez et al. 2005), another online catalogue for Brazil (Morales and Marinoni 2017), and an extensive taxonomic study of the flower flies of Suriname (Reemer 2010, 2014, 2016). In addition, Thompson et al. (2010) gave a very comprehensive synopsis of the Central American Syrphidae.

Thompson et al. (2010) stated that ca. 1,800 flower fly species are described from the Neotropical Region, but other authors argue that this may be only half of the actual number of species (Reemer 2016). Thus, Ecuadorian syrphid fauna comprises roughly 11.2% of the described Neotropical species. Emulating the arguments of Reemer (2016), the syrphid fauna of Ecuador might be two to four times larger, up to 900 species, if we compare the known species of other taxa in this country with the total number of species in the Neotropical Region. Cárdenas et al. (2009) estimated that Ecuador has 16.3% of the Neotropical species of the family Tabanidae (Diptera). Mittermeier et al. (2005) calculated that the bird species present in Ecuador are ca. 47% of the total number of species in the Neotropics. With an estimate of 4,000 species of butterflies (Salazar and Donoso 2014, M. Espeland pers. comm.), Ecuador probably hosts half of the Neotropical diversity of this order. In other words, considering these numbers and the fact that Syrphidae is underexplored in Ecuador (Amorim 2009), we are far from having a good estimate of the total number of flower fly species for Ecuador.

We think that the inventory and study of the Syrphidae fauna are essential not only to describe new species from Ecuador, but also to help in the selection of areas to protect, based on species richness, and to improve the management of conservation areas in this country. Salazar and Donoso (2014) mentioned that the taxonomic complexity, the lack of experts for some groups, the high species richness, and the endemicity of many invertebrates in Ecuador make the study of its invertebrate fauna a major challenge in science. Moreover, Ecuador has two biodiversity hotspot regions: Tropical Andes and Tumbes-Chocó-Magdalena (Myers et al. 2000, Mittermeier et al. 2004). These regions are heavily threatened and need urgent conservation efforts. In such cases, faunistic studies should have priority to understand the biological diversity of those hotspots. Furthermore, the poor knowledge of the relationships between flower flies and their prey, as well as the unknown associations with host plants, make the study of this group essential 1) to improve our understanding about their roles in the ecosystem performance and organic matter decomposition, 2) to evaluate the biological richness of Ecuador in order to establish new management and control protocols over its natural resources, and 3) to revise the quarantine and international trade policies for preventing potential pest species dispersal and creating new banned species list.
